# Case Report Demonstrating Multifactorial Risks of Anterior Cruciate Ligament Re-tear Injuries and Appropriate Response Among Those With High Chance of Recurrence

**DOI:** 10.7759/cureus.24965

**Published:** 2022-05-13

**Authors:** Jorge Perera, Mark D Miller, Paul Danahy

**Affiliations:** 1 Orthopedic Surgery, Lake Erie College of Osteopathic Medicine, Bradenton, USA

**Keywords:** anterior cruciate ligament (acl), functional outcome of acl reconstruction, acl rehabilitation, anterior cruciate ligament (acl) reconstruction, acl injury

## Abstract

We present the case of a collegiate football player with an extensive bilateral ligamentous knee injury history to elucidate the mechanisms and possible explanations behind why some athletes sustain recurrent injuries. We hope to initiate thought on altering rehabilitation schedules for athletes who are at an increased risk of re-injury. A 21-year-old collegiate American football player presented with a re-tear of his left anterior cruciate ligament (ACL) and medial meniscus following reconstructive surgery. The initial injury occurred to the patient when he was 15 and suffered a right ACL, lateral collateral ligament, and lateral meniscus tear in a non-contact injury. At the age of 19, he suffered his second injury, a contact-associated left ACL tear. Upon return to play six months following the left ACL tear, the patient sustained a non-contact bucket handle tear of the right medial meniscus. One year later, he presented with a re-tear of his left ACL. His initial left and right ACLs were repaired with hamstring autografts, and his current left ACL was repaired with a bone-patellar tendon-bone graft. This case illustrates an all too common situation plaguing the modern orthopedic sports medicine surgeon. At what point should a surgeon diverge from the standard rehabilitation schedule of ACL surgery due to a patient being at too high of a risk for a re-tear? We propose further investigations into risk factors as well as rehabilitation protocols to help surgeons identify and optimize treatment for these patients.

## Introduction

Soft tissue knee injuries continue to plague athletes across the nation. From the trivial weekend warrior to the elite professional star, feeling a “pop” in the knee remains one of the great fears of today’s athletes. The anterior cruciate ligament (ACL) is one of the four major ligaments that primarily stabilizes the knee joint [1]. The ACL primarily prevents anterior sliding of the tibia relative to the femur, as well as provides protection to the menisci from the shearing forces that occur during high-impact athletic movements [1]. We present the case of a collegiate football player with an extensive bilateral ligamentous knee injury history hoping to elucidate the mechanisms and possible explanations behind why some athletes sustain several ligamentous knee injuries. In this particular case, the patient sustained four catastrophic knee injuries within a span of five years. As his history is unraveled, it quickly becomes apparent why the risk of ACL injury is widely considered to be multifactorial [1]. In addition, we examine the standard rehabilitation timeline of ACL surgery and inquire about when the accumulation of patient risk factors requires a deviation from the routine schedule.

## Case presentation

Case

A 21-year-old collegiate football player presented with a re-tear of his left ACL and (medial) meniscus following reconstructive surgery with a hamstring graft one year prior. This was his fourth knee surgery in the past five years.

History

The patient suffered the first catastrophic knee injury at the age of 15 in August 2011. It was a non-contact injury during a high school football practice as the patient jumped for a ball and landed awkwardly on his right knee. He stated that he felt a “pop” and as if his tibia shifted laterally on his femur when he landed. The knee became swollen immediately upon injury. Lachman testing was inconclusive due to resistance, and the patient could put minimal weight on his right leg. Magnetic resonance imaging (MRI) results stated a diagnosis of a complete rupture of the right ACL, lateral meniscus, and lateral collateral ligament (LCL). Due to lack of mobility and severe inflammation, the surgery was delayed one month while physical therapy reduced the swelling and improved range of motion post-injury. The ACL was repaired using a hamstring graft from the patient, and the LCL was repaired using an allograft. The patient performed physical therapy for six months at a local rehabilitation center and was cleared for full participation in July 2012 with a knee brace on his right knee.

**Figure 1:**
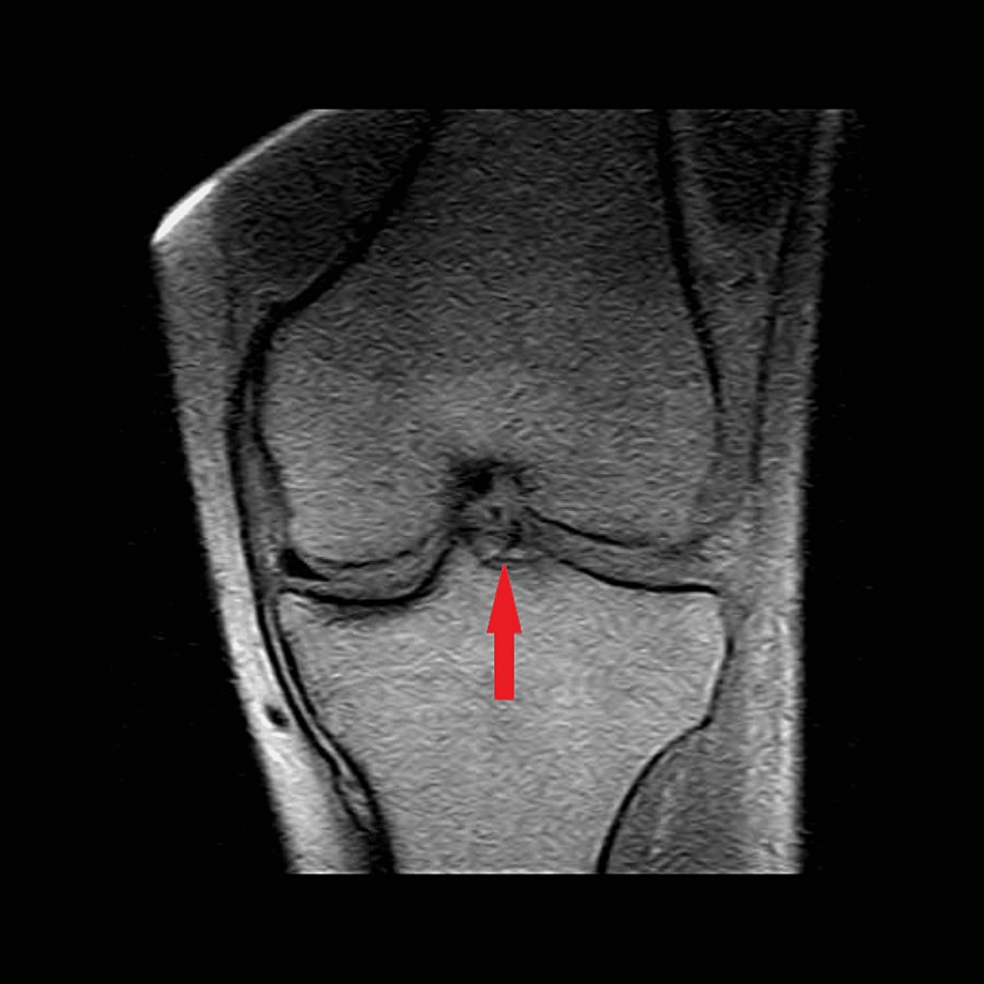
T1 coronal MRI left knee. Red arrow points toward the evidence of ligament damage. MRI: magnetic resonance imaging

**Figure 2:**
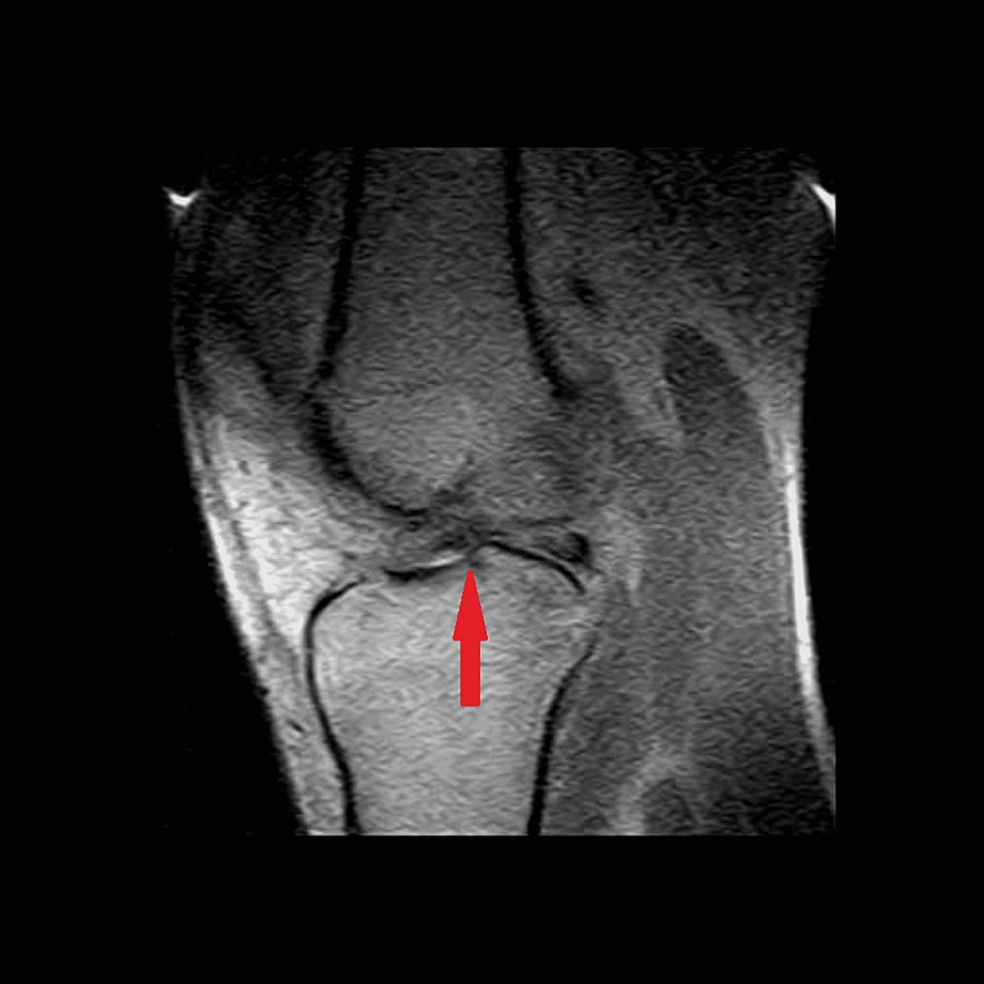
T1 sagittal MRI of the left knee. Red arrow points toward the evidence of inflammation. MRI: magnetic resonance imaging

**Figure 3:**
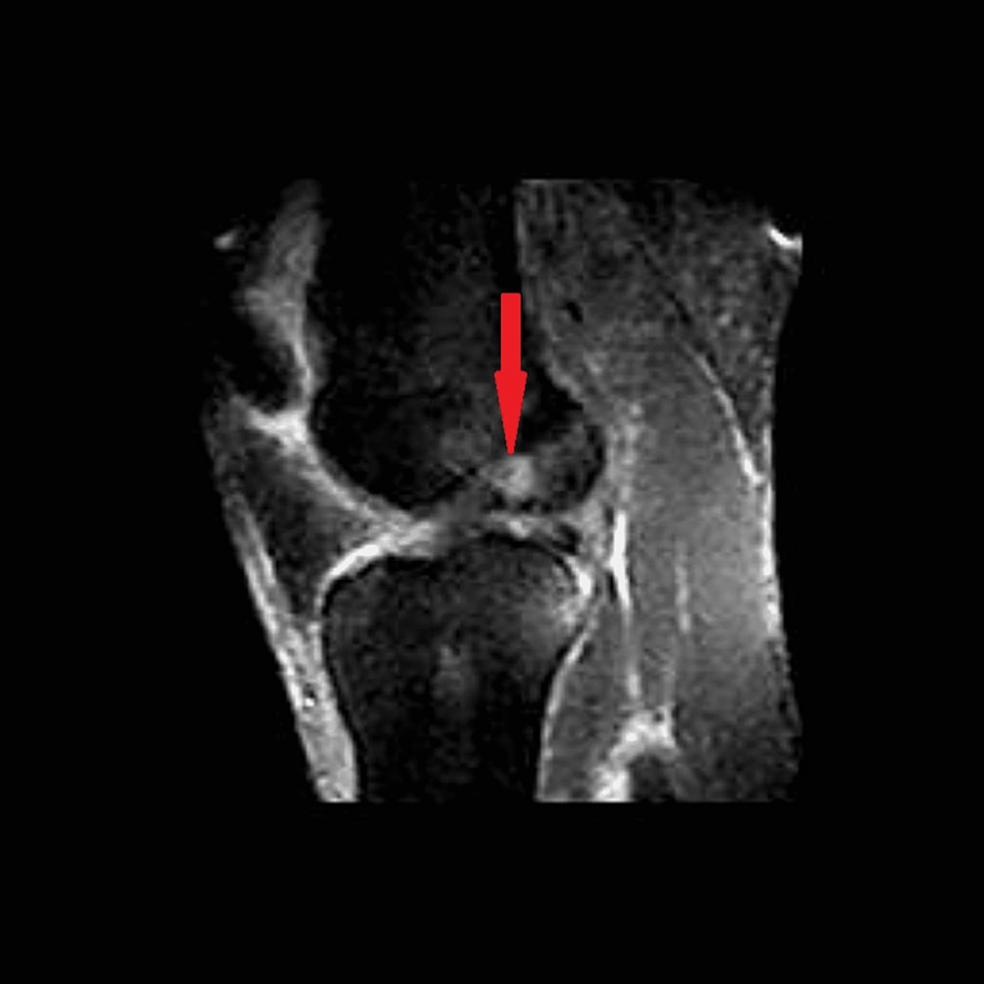
T2 sagittal STIR left knee. Red arrow points toward the evidence of inflammation. STIR: short TI inversion recovery

In September 2012, the patient suffered another non-contact knee injury but this time to his left knee. While running under a pass intended for him, his left knee “gave out medially” when attempting to quickly change direction. He felt a similar “pop” to the one he felt one year prior. However, his left knee did not swell to the extent his right knee did. On MRI, a diagnosis of a complete ACL rupture, a partial tear of the medial collateral ligament, and a radial tear of the lateral meniscus was given. Surgery was withheld once again to regain range of motion and reduce the inflammation. On the six-week follow-up visit, the patient stated that he felt much better with minimal pain. Strength was equal bilaterally with a negative Lachman and posterior drawer test, as well as a negative medial and lateral stress test. There was minimal inflammation and the patient retained full range of motion of the knee. The decision was made that the MRI analysis may have been an overdiagnosis (Figures 1-3), and the patient was allowed to return to play.

In April 2015, the patient presented with a contact left knee injury as a freshmen in college. While engaged in a block, the patient planted his left cleat into the turf and felt a distinct pop and intense pain as his knee displaced medially. He stated this was the most painful knee injury he had endured. While on the field, the training staff was able to perform an easily positive Lachman test. The MRI confirmed the diagnosis of a torn left ACL and medial meniscus. Such as with the first surgery, the decision was made to move forward with a hamstring graft surgery. There were no complications, and the surgery was a success. The patient began physical therapy during the summer with a plan to return to play the next spring.

In October of the same year, while performing a stretch of his lower right extremity, the patient felt another “pop,” but this time back in his right knee. The patient stated it was not nearly as painful as his previous injuries but he did walk with a limp. MRI confirmed a bucket handle tear of the right medial meniscus. Arthroscopy was performed and removal of the meniscus was completed successfully. The patient was allowed to walk that day and was still on track to participate in spring football with a knee brace on his left knee only.

In the spring of 2016, the patient presented with a “torn left ACL” after the spring football game. He stated that he made a tackle and despite the brace being there, he felt his knee buckle underneath him. Due to his previous history, the patient stated he knew the feeling of a torn ligament and was convinced he had suffered yet another tear, this time of his surgically repaired left ACL. He stated the pain was not nearly as significant as his other injuries. MRI confirmed the presence of a complete rupture of the ACL. The patient agreed to move forward with bone-patellar bone graft repair of his left ACL and was advised to no longer participate in football activities.

Outcome

Despite this advice, the patient is able to participate in a limited role during his senior year of collegiate football. Since his last surgery in 2016, the patient has not sustained any re-injury of his ligaments or menisci and is able to fully participate in different exercise programs, though he no longer plays football or any sport that requires quick changes of direction.

## Discussion

The number of ACL-related injuries in athletes younger than 18 has increased in the past two decades with a multitude of theories and likely reasons. These include the increasing number of children participating in sports, increased training leading to more explosive athletes, and increased diagnostic resources [1]. With a direct blow to the knee, the extrinsic force applied to the ligaments of the joint can quite obviously perturb normal mechanics and lead to rupture. Non-contact injuries deal with more intrinsic forces and are therefore harder to pinpoint the exact cause of ligament tearing. Video analysis of ACL injuries portrays a common body position associated with a non-contact ACL rupture; the internally rotated hip, knee in near full extension, a planted foot, and a decelerating body creating valgus collapse of the knee [1]. Aside from vulnerable positioning, multiple factors such as genetics, hormone levels, and body mass index (BMI) likely play a role in the damage to internal knee structures. However, none are as strong predictors for ACL injury as the presence of a previous ACL tear. In fact, one study found the rate of re-injury in athletes with a history of ACL reconstruction to be 15 times greater than those in the control group [2].

Annually, the highest average rate of ACL injury for men was in American football (0.17 per 1,000 athlete-exposure), while the highest average annual rate of ACL injury for women was found in lacrosse [3]. It is generally accepted that females tear their ACLs more often than males. This can be attributed to the female skeletal anatomy, with a wider pelvis contributing to the naturally vulnerable valgus position of the knee [4]. This provides an example of how non-modifiable risk factors can contribute to a higher risk of injury. By the same token, age also plays a role in re-tear rates. The patient was 15 at the time of his first injury, despite high-school athletes having lower overall rates of ligament tear than collegiate athletes [1]. However, the younger an athlete suffers an ACL injury, the higher the chance of recurrence [1]. Acknowledging the increased risk certain variables bestow upon patients is important to recognize.

Of these, non-modifiable risk factors are important to not discount. Four of such have been identified and studied extensively, including increased posterior tibial slope, increased middle cartilage slope, decreased lateral meniscus height in the posterior compartment in females, and a decrease in meniscal bone angle in males [5]. Due to the interaction of these different anatomic variations, when an axial compressive force is applied to the knee joint, an anterior shear force and an internal tibial torque are produced. It is well accepted that anterior tibial translation and internal tibial rotation increase ACL injury risk, displaying why the geometry of one’s internal knee structures is of the utmost importance [6]. Of equal importance is incorporating the modifiable with non-modifiable risk factors when establishing a prophylactic plan for reducing re-injury. An increase in BMI will increase the axial force applied to an anatomically susceptible knee, leading to an increased risk of injury [5]. Knowing the patient’s anatomy in combination with risk factors that are within their control can be the difference in a successful return to the court or field.

Speaking of, one theory that has garnered much publicity is the idea that artificial turf is much more dangerous for ligament injuries compared to competing on natural grass. A study that examined data from 2004 to 2009 through the National Collegiate Athletic Association Injury Surveillance system seems to support this train of thought [7]. There was an incidence rate of 1.73 ACL injuries per 10,000 football athlete-exposure on artificial playing surfaces compared with a rate of 1.24 per 10,000 on natural grass. The rate of ACL injury was found to be 1.39 times higher than the injury rate found on real grass [7]. In addition, players were 10.09 times more likely to sustain an ACL injury during competition when compared with practices [7]. In this specific case, the patient tore his ACL on natural grass and during practice in high school, making this one of the rarer cases. He also experienced a tear on artificial turf during a spring football game, placing his last injury in the more common category of ACL injury. What else, then, could have played a role in this patient’s recurrent injuries?

Possibly the first thought in many minds when speaking of re-tears is the discussion of which type of graft was first used. The consensus remains that ACL graft choice should be based on the surgeon’s comfort level and specific patient characteristics. With this said, slight variations in data between the outcomes of certain grafts do exist. For example, a recent meta-analysis that compared bone-patellar tendon-bone (BPTB) autograft versus hamstring autograft in over 47,000 patients found a 2.84% hamstring tear rate compared to 2.80% in BPTB [8]. Of course, many more factors have been proposed to increase the re-tear rate. Kaeding et al. found a 4.4% graft re-tear rate which was positively associated with younger age, higher Marx activity level score, and use of allograft [9]. Maletis et al. found no significant difference between the failure rates of allografts versus hamstring autografts, although the average patient age for the autograft group was 24 while the allograft group was 35 [10]. This plays an important role as it is well known that younger, highly active patients have a significantly higher re-tear rate with allografts, with a rate as high as 25% being reported [8]. Ultimately, patient dynamics should be a determining factor when deciding which graft to use. In this case, for an adolescent with open growth plates, hamstring autograft remains the preferred choice, with BPTB being recommended for other age groups [8].

Regardless of which graft was used, the vast majority of physical therapy before and after the injury has been historically focused on strength and range of motion. This goal-oriented type of physical therapy is the norm for younger non-professional athletes. However, there may be a crucial difference between this form of neuromuscular training compared to a smarter, more effective type utilized by the best physical therapists competitive sports have to offer. Despite the fact that ACL tearing occurs too quickly for reflexive muscular activation, athletes can still “pre-program” their knee to avoid dynamic valgus during unexpected pivots or loads during competition [11]. Prospective cohort studies have investigated the differences in athlete outcomes who utilized plyometrics, balance, stretching, or a comprehensive approach when rehabilitating postoperatively. Through a systematic examination of the data, it was determined that programs that incorporated plyometrics combined with feedback regarding proper form were more likely to reduce re-injury rates as opposed to the standard [12]. In addition, programs that also incorporated strength training with proper loading and landing techniques displayed even more effective results in reducing the risk of re-injury [12]. The type of therapy received postoperatively may be just as imperative as a quality surgery in determining a successful return to play.

Once back on the field, an important question looms over many athletes’ heads: brace or no brace? Functional bracing has been researched with inconclusive results. In a study performed by McDevitt et al., randomized cohorts were placed either in the braced or control group following ACL reconstructive surgery. The braced group used their functional knee brace for all jumping, pivoting, and cutting activities for one year while the control group was allowed to advance through training protocols without a brace. Ultimately, the researchers found no differences between the groups in knee stability, range of motion, or strength [13]. The data were insufficient in determining whether the brace reduced the risk of re-injury. The last injury sustained by the patient was with a knee brace on, further challenging the usefulness of knee brace in injury prevention.

## Conclusions

The most pertinent risk factor for ACL injury is the history of prior injury. Instead of the basic guidelines handed down for all non-professional athletes regarding their surgery and recovery, there may be more orthopedic surgeons and physical therapists can do to improve the return to play rates for injury-prone athletes. Understanding the particular patient’s anatomy in combination with their modifiable risk factors can drastically impact athletes’ susceptibility to future injury. Graft use that provides the statistically best outcomes should be based on patient demographics and their desire for knee functionality postoperatively. Physical therapy that emphasizes education on proper body positioning and landing form while including plyometric-based strength training should become the norm in rehabilitation. Regular follow-up and continued support throughout the return to sports period must be prioritized. The standard timetable for a return to play following an ACL tear should be individualized with a benchmark approach that implements certain educational and physical parameters that patients must reach before being allowed back on the playing field.
